# Selinexor enhances the sensitivity of hepatocellular carcinoma cells to sorafenib by regulating the BAX/Bcl-2/PUMA apoptotic pathway and the XPO1/p27 cell cycle pathway

**DOI:** 10.3389/fonc.2026.1762822

**Published:** 2026-03-06

**Authors:** Shenghong Du, Ling Wang, Chen Chen, Yu Sun, Qian Liu, Jingfei Shi, Feng Zhang, Kai Wang, Chao Cui

**Affiliations:** 1 Department of Hematology, The Affiliated Taian City Central Hospital of Qingdao University, Taian, Shandong, China; 2 Postdoctoral Research Station, Shandong Boaoke Biotechnology Co., Ltd., Liaocheng, Shandong, China; 3 Department of Hepatology, Qilu Hospital of Shandong University, Jinan, China; 4 Department of Infectious Disease, Qilu Hospital of Shandong University Dezhou Hospital, Dezhou, China; 5 Department of Pediatrics, Taian Eightty-Eight Hospital, Taian, China; 6 Department of Clinical and Basic Medicine, Shandong First Medical University, Jinan, Shandong, China

**Keywords:** apoptosis pathway, cell cycle pathway, drug sensitivity, hepatocellular carcinoma, selinexor, sorafenib

## Abstract

**Introduction:**

Sorafenib remains the first-line targeted therapy for advanced hepatocellular carcinoma (HCC), but its clinical efficacy is severely limited by intrinsic and acquired drug resistance. Dysregulation of the BAX/Bcl-2/PUMA apoptotic pathway and XPO1/p27 cell cycle pathway is closely associated with sorafenib resistance. This study aimed to explore whether selinexor, a selective nuclear export inhibitor, could enhance the sensitivity of HCC cells to sorafenib and to clarify the underlying molecular mechanism.

**Methods:**

A series of *in vitro* cell experiments (Huh7, SK-HEP-1, HepG2) and *in vivo* Huh7 xenograft nude mouse models were conducted. CCK-8 assay, flow cytometry, Western blot and immunohistochemistry were used to detect cell proliferation, cell cycle distribution, apoptosis, and the expression levels of key proteins related to apoptosis and cell cycle pathways. The additive effect of the drug combination was verified by comparing the experimental inhibitory rate with the theoretical additive effect.

**Results:**

Selinexor combined with sorafenib significantly inhibited tumor growth in nude mice, with a stronger inhibitory effect than monotherapy. *In vitro*, the two drugs exerted an additive effect on suppressing the proliferation of Huh7, SK-HEP-1 and HepG2 cells. Meanwhile, the combination treatment induced obvious G1 phase arrest in Huh7 cells and markedly increased the apoptosis rate of Huh7 and HepG2 cells. Mechanistically, the combined therapy upregulated the expression of pro-apoptotic proteins BAX and PUMA as well as cell cycle regulator p27, while downregulating anti-apoptotic protein Bcl-2 and nuclear export protein XPO1.

**Discussion:**

This study confirms that selinexor enhances the sensitivity of HCC cells to sorafenib by regulating the BAX/Bcl-2/PUMA apoptotic pathway and the XPO1/p27 cell cycle pathway. The combination strategy provides a novel potential approach for improving the therapeutic efficacy of sorafenib and overcoming both intrinsic and acquired sorafenib resistance in HCC. The main limitations of this study are the lack of RT-PCR verification and further detection of downstream apoptotic effector molecules, which need to be explored in future research.

## 1 Introduction

Hepatocellular carcinoma (HCC) is one of the malignant tumors with extremely high incidence and mortality worldwide, and targeted therapy has become an important treatment modality for advanced HCC (1). As a multi-kinase inhibitor, sorafenib exerts antitumor effects by inhibiting tumor angiogenesis and cell proliferation signaling pathways, and serves as a first-line targeted therapeutic agent for advanced HCC. However, the frequent development of drug resistance in clinical practice severely limits its therapeutic efficacy (2). This resistance is mainly divided into two types: intrinsic resistance (primary resistance), in which tumors do not respond to sorafenib from the outset, and acquired resistance (secondary resistance), in which tumors initially respond but eventually develop resistance during treatment. Tumor cell apoptosis escape and cell cycle dysregulation are key mechanisms underlying sorafenib resistance, among which the abnormal regulation of the Bcl-2 associated X protein (BAX)/B-cell lymphoma 2 (Bcl-2)/p53 upregulated modulator of apoptosis (PUMA) apoptotic pathway and Exportin 1 (XPO1)/Cyclin-dependent kinase inhibitor 1B (p27) cell cycle pathway is particularly critical (3–7). As pro-apoptotic proteins, BAX and PUMA, together with the imbalanced expression of the anti-apoptotic protein Bcl-2, directly affect the apoptotic sensitivity of tumor cells (8–10). The nuclear export protein XPO1 can mediate the nuclear export of the cell cycle inhibitor p27 and accelerate its degradation, leading to cell cycle dysregulation and abnormal proliferation, which are important molecular bases for the occurrence, development, and drug resistance of HCC (11, 12).

Selinexor, as the first selective inhibitor of nuclear export, specifically inhibits the function of XPO1, blocks the nuclear export of tumor suppressor proteins, and promotes their accumulation in the nucleus to exert antitumor effects (13). Clinically, selinexor-based quadruple combination therapy (with palbociclib, pembrolizumab, and cord blood NK cells) achieved partial response in a metastatic advanced HCC patient, reducing liver and lung metastases by ~60% and ~90%, respectively (14). Mechanistically, selinexor reduces intranuclear HIF-1α levels in hepatoma cells via XPO1/CRM1 inhibition, thereby enhancing radiosensitivity (15). Recent studies have shown that selinexor can enhance the sensitivity of various tumor cells to chemotherapeutic drugs and targeted agents. For example, selinexor has been reported to combine with gemcitabine-nab-paclitaxel to inhibit tumor cell growth, colony formation, and spheroid formation, while extending mouse survival by perturbing pancreatic ductal adenocarcinoma-supporting signaling networks and depleting CD44^+^ cancer stem cell populations (16). In high-risk neuroblastoma, co-treatment with selinexor and the Aurora Kinase A (AURKA) inhibitor alisertib has been shown to modify p53 activity through dual actions: blocking XPO1-dependent nuclear export of p53 and inhibiting AURKA-induced p53 degradation, with subsequent effects on p53-mediated cellular responses and treatment outcomes (17). However, whether selinexor can enhance sorafenib sensitivity in HCC by modulating the BAX/Bcl-2/PUMA apoptotic pathway and XPO1/p27 cell cycle pathway—either as a combination therapy to improve primary efficacy or as a strategy to overcome intrinsic/acquired resistance—and the specific underlying mechanism, remains unclear. In this study, *in vitro* and *in vivo* experiments were conducted to investigate the antitumor efficacy of the combination of selinexor and sorafenib, with a focus on clarifying its regulatory effects on the BAX/Bcl-2/PUMA apoptotic pathway and XPO1/p27 cell cycle pathway, thereby providing experimental evidence for a novel combination therapy strategy and a potential solution for overcoming both intrinsic and acquired sorafenib resistance in HCC.

## 2 Materials and methods

### 2.1 Cell lines and reagents

Huh7, SK-HEP-1, and HepG2 HCC cell lines were routinely cultured in DMEM medium supplemented with 10% fetal bovine serum and incubated in a constant-temperature incubator at 37°C with 5% CO_2_. Selinexor was purchased from Selleck Chemicals, and sorafenib was obtained from MedChemExpress (MCE). Matrigel matrix was purchased from BD Biosciences, and the CCK-8 kit was from Biosharp. Antibodies used for Western blot analysis included BAX (Santa Cruz), Bcl-2 (Santa Cruz), p27 (Santa Cruz), XPO1 (BD Biosciences), and PUMA (Santa Cruz). Antibodies for immunohistochemical staining were BAX (ABclonal), Bcl-2 (ABcam), p27 (ABcam), and XPO1 (ABclonal).

### 2.2 Animal experiments

Six-week-old female nu/nu athymic nude mice were purchased from Hubei Provincial Center for Disease Control and Prevention. After 1 week of adaptive feeding, 5×10^6^ Huh7 cells (suspended in 0.15 mL high-concentration Matrigel) were subcutaneously injected into the right dorsal region to establish xenograft tumor models. Once tumors formed, the mice were randomly divided into 4 groups (n=5 per group): blank control group (administered with Pluronic F-68/PVP-K29/32), selinexor monotherapy group (Sel-10, 10 mg/kg), sorafenib monotherapy group (Sor-10, 10 mg/kg), and selinexor + sorafenib combination group (Sel-10 + Sor-10, 10 mg/kg selinexor + 10 mg/kg sorafenib). The doses of selinexor and sorafenib (10 mg/kg each), as well as the 4-week treatment duration, were all determined according to preliminary data from our laboratory. These preliminary studies demonstrated that this dose regimen achieved stable and significant tumor growth inhibition while inducing minimal acute toxicity (e.g., no significant body weight loss or grade ≥2 adverse events) in a pilot Huh7 HCC xenograft model, thus balancing therapeutic efficacy and animal welfare. The application of identical individual doses (10 mg/kg for each drug) in the combination group was designed to eliminate confounding variables caused by dose adjustment, enabling a direct and unbiased comparison of antitumor efficacy between monotherapy and combination therapy, and to accurately verify the additive effect of the two drugs rather than a dose-dependent effect. Treatments were initiated 1 day after cell implantation, and drugs were administered via oral gavage 3 times per week for 4 consecutive weeks. Tumor length, width, and thickness were measured with calipers 3 times weekly, and tumor volume was calculated using the formula (length × width × thickness). Changes in mouse body weight and adverse reactions were recorded. Body weight was measured on d1, d7, d11, d14, and d17, and adverse reactions (including diarrhea, lethargy, and loss of appetite) were monitored daily and scored according to the Common Terminology Criteria for Adverse Events (CTCAE) v5.0. On day 17, mice were euthanized, and tumor tissues were dissected. After weighing, part of the tissues was fixed in 10% formalin for immunohistochemical detection, and the remaining tissues were reserved for subsequent experiments.

### 2.3 Cell proliferation assay

The CCK-8 assay was used to evaluate the effect of drugs on HCC cell proliferation. Huh7, SK-HEP-1, and HepG2 cells in the logarithmic growth phase were seeded into 96-well plates at a density of 5×10³ cells per well. After 24 h of incubation, different concentrations of selinexor or sorafenib were added. Following further incubation for 24 h, 48 h, or 72 h, 10 μL of CCK-8 reagent was added to each well, and the plates were incubated for an additional 2 h. The absorbance at 450 nm was measured using a microplate reader, and the cell proliferation inhibition rate was calculated. Cells were assigned to four treatment groups: control, sorafenib monotherapy, selinexor monotherapy, and the combination of selinexor and sorafenib. Cell proliferation was detected by the CCK-8 assay after 48 h or 72 h of treatment. The half-maximal inhibitory concentration (IC_50_) values were calculated using GraphPad Prism 9.0 software with a four-parameter logistic regression model (log (agonist) vs. response, variable slope). To distinguish between additive and synergistic effects, the theoretical additive proliferation inhibition rate of the combination treatment was calculated using the formula: Theoretical additive rate = Rate of selinexor monotherapy + Rate of sorafenib monotherapy - (Rate of selinexor monotherapy × Rate of sorafenib monotherapy). The experimental inhibition rate of the combination treatment was then compared with the theoretical additive rate. A significant difference between the experimental and theoretical rates indicates a synergistic or antagonistic effect, while no significant difference indicates an additive effect. The concentrations of selinexor and sorafenib used in all *in vitro* cell experiments were selected based on the results of preliminary CCK-8 proliferation assays. Specifically, we chose concentrations that achieved 20%-40% cell growth inhibition (IC_20_-IC_40_) as single agents, a widely accepted range for evaluating drug-drug interactions in combination therapy studies. This concentration range ensures that the effects of each drug are detectable but not maximal, thereby avoiding false-negative or false-positive results in the assessment of additive effects.

### 2.4 Cell cycle analysis

Huh7 and HepG2 cells were divided into blank control group, selinexor group, sorafenib group, and selinexor + sorafenib combination group. After 72 h of drug treatment, cells were harvested, washed twice with pre-cooled PBS, fixed in 70% ethanol overnight, and stained with PI staining solution for 30 min at room temperature in the dark. Cell cycle distribution was detected by flow cytometry, and the results were analyzed using ModFit software.

### 2.5 Cell apoptosis assay

After 48 h of drug treatment, cells in the above groups were collected, stained with Annexin V-FITC/PI apoptosis detection kit solution, and incubated for 15 min at room temperature in the dark. The apoptotic rate was detected by flow cytometry, and the proportions of early and late apoptotic cells were analyzed.

### 2.6 Immunohistochemical staining

Tumor tissues were paraffin-embedded, sectioned, and dewaxed to water. Antigen retrieval was performed using citrate buffer, and endogenous peroxidase activity was blocked with 3% H_2_O_2_. After blocking, sections were incubated with primary antibodies against BAX, Bcl-2, p27, and XPO1 at 4°C overnight, followed by incubation with secondary antibody at room temperature for 30 min. Diaminobenzidine (DAB) was used for color development, and sections were counterstained with hematoxylin, dehydrated, cleared, and mounted. Images were captured and observed under a microscope.

### 2.7 Western blot analysis

After Huh7 cells were treated with different drugs for 72 h, total proteins were extracted, and protein concentration was determined by the BCA method. Equal amounts of proteins were subjected to SDS-PAGE electrophoresis and transferred onto PVDF membranes. Following blocking, the membranes were incubated with primary antibodies against BAX, Bcl-2, p27, XPO1, PUMA, and the internal reference GAPDH at 4°C overnight, then with fluorescent secondary antibodies at room temperature for 1 h. Protein bands were visualized using an ECL chemiluminescence kit, and the gray values of bands were quantitatively analyzed by ImageJ software.

### 2.8 Statistical analysis

All data were analyzed using SPSS 22.0 statistical software. Quantitative data were presented as mean ± standard deviation (*x* ± s). One-way analysis of variance (ANOVA) was used for comparisons among multiple groups, and LSD-*t* test was performed for pairwise comparisons. A *P* value <0.05 was considered statistically significant.

## 3 Results

### 3.1 Combined use of selinexor and sorafenib significantly inhibits xenograft tumor growth in nude mice

**Figure 1:**
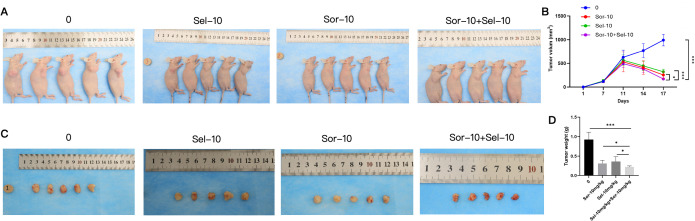
Inhibitory effect of combined selinexor and sorafenib on xenograft tumor growth in nude mice. **(A)** Photographs of final tumor volumes from different treatment groups. **(B)** Quantitative bar graph of final tumor volumes from different treatment groups. **(C)** Photographs of the final tumor weights from different treatment groups. **(D)** Quantitative bar graph of the final tumor weights from different treatment groups. (mean ± SD, n=5; Sel-10 = selinexor monotherapy (10 mg/kg), Sor-10 = sorafenib monotherapy (10 mg/kg), Sel-10 + Sor-10 = selinexor + sorafenib combination (10 mg/kg + 10 mg/kg); *P < 0.05, **P <0.01, ***P <0.001. Statistical analysis was performed using one-way ANOVA followed by LSD-*t* test). (Refer to **Table 1** for detailed measured tumor volume data at key time points during the entire treatment period).

**Figure 2:**
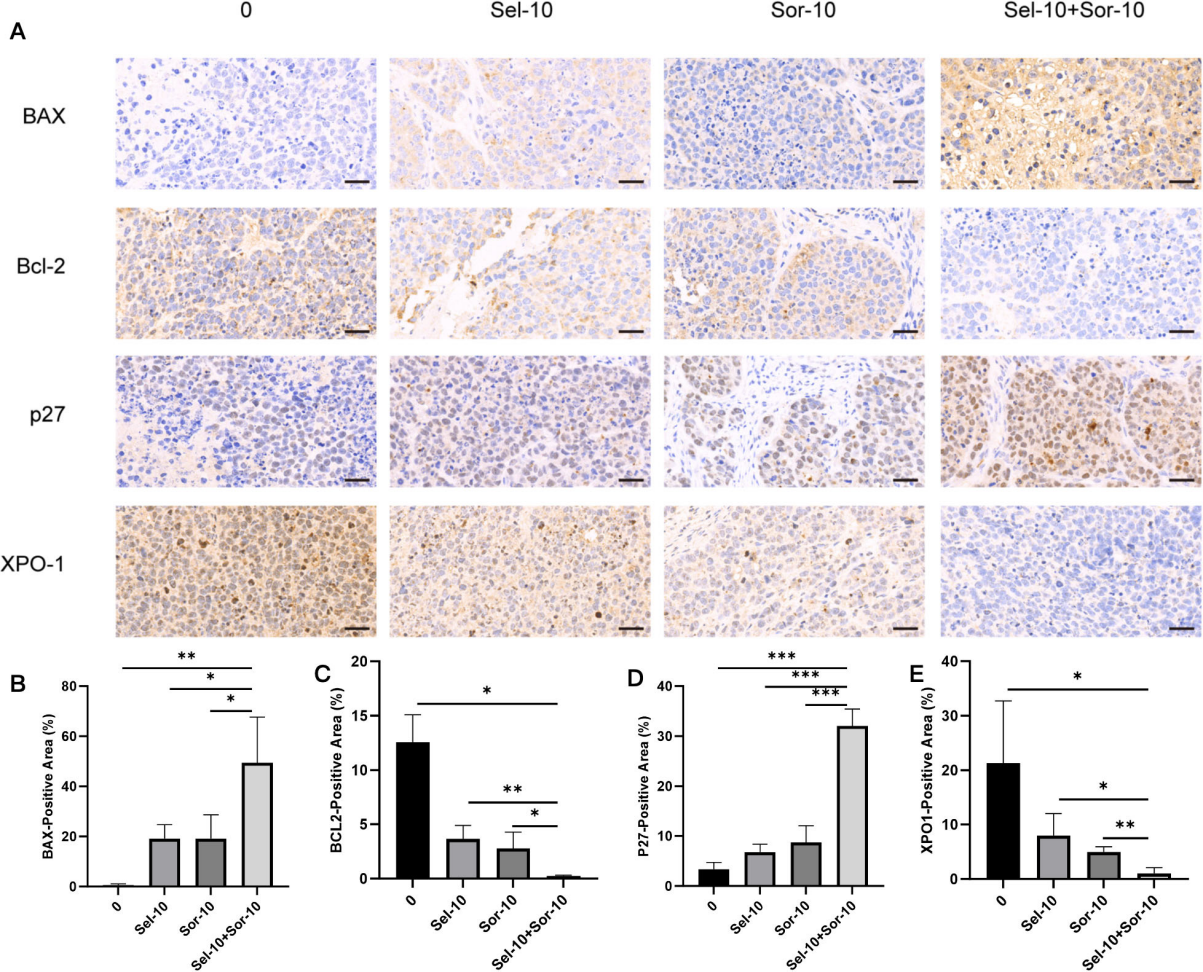
Immunohistochemical detection results of related proteins (BAX, Bcl-2, p27, and XPO1) in xenograft tumor tissues of nude mice from different treatment groups. **(A)** Representative immunohistochemical images of BAX, Bcl-2, p27, and XPO1 expression (magnification=40×; scale bar=50 μm). **(B)** Quantitative analysis of BAX expression among treatment groups. **(C)** Quantitative analysis of Bcl-2 expression among treatment groups. **(D)** Quantitative analysis of p27 expression among treatment groups. **(E)** Quantitative analysis of XPO1 expression among treatment groups. (Quantitative analysis of immunohistochemical staining intensity: mean ± SD, n=5 fields per section; Sel-10 = selinexor monotherapy (10 mg/kg), Sor-10 = sorafenib monotherapy (10 mg/kg), Sel-10 + Sor-10 = selinexor + sorafenib combination (10 mg/kg + 10 mg/kg); *P < 0.05, **P <0.01, ***P <0.001. Statistical analysis was performed using one-way ANOVA followed by LSD-*t* test). Coordinated modulation of BAX and Bcl-2 in tumor tissues is consistent with *in vitro* PUMA upregulation, supporting the functional activation of the BAX/Bcl-2/PUMA apoptotic pathway *in vivo* (refer to Section 3.1 and Discussion for details).

Animal experiment results showed that the tumor volume and weight in each drug treatment group were significantly lower than those in the blank control group (P<0.05), and the inhibitory effect was most prominent in the selinexor + sorafenib combination group. The final tumor volume and weight were 966.9 mm³ and 0.926 g in the blank control group, 274.8 mm³ and 0.314 g in the Sel-10 group, 305.0 mm³ and 0.364 g in the Sor-10 group, and reduced to 169.2 mm³ and 0.216 g in the Sel-10 + Sor-10 group, respectively (Volume: P < 0.001 vs. control group; P < 0.001 vs. Sel-10; P < 0.05 vs. Sor-10 monotherapy groups; Weight: P < 0.001 vs. control group; P < 0.05 vs. Sel-10 ; P < 0.05 vs. Sor-10 monotherapy groups) (**Figures 1A, C**, **Table 1**). Quantitative bar graphs in **Figures 1B, D** intuitively illustrate the final tumor volume and weight across all groups, respectively, with significant differences between groups clearly indicated. In addition, **Table 1** presents the serial tumor volume measurements at key time points (D1, D7, D11, D14, D17) for each treatment group. Collectively, these endpoint and time-course data provide a rigorous and comprehensive characterization of the dynamic antitumor efficacy of the tested regimens. Regarding adverse reactions, mild diarrhea (CTCAE grade 1) was observed in 2 mice in the selinexor group and 1 mouse in the combination group, which resolved spontaneously without additional treatment. No lethargy, loss of appetite, or other severe adverse reactions were observed in any group. Immunohistochemical results demonstrated that the protein expression levels of BAX and p27 were significantly upregulated in the selinexor and sorafenib monotherapy groups, and the upregulation was further enhanced in the combination group (BAX: P < 0.01 vs. control group; P < 0.05 vs. both monotherapy groups;p27: P<0.001 vs. control group; P < 0.001 vs. both monotherapy groups). In contrast, the expression of Bcl-2 and XPO1 was significantly downregulated in the combination group compared with the control group and monotherapy groups (Bcl-2: P < 0.05 vs. control group; P < 0.01 vs. Sel-10; P < 0.05 vs. Sor-10; XPO1: P<0.05 vs. control group; P < 0.05 vs. Sel-10; P < 0.01 vs. Sor-10) (**Figure 2**). Notably, the coordinated upregulation of BAX and downregulation of Bcl-2 in xenograft tumor tissues are functionally consistent with our *in vitro* Western blot data showing a 4.90-fold upregulation of PUMA in the combination group. As PUMA is a well-characterized upstream regulator that bridges p53-dependent signaling to BAX activation and Bcl-2 neutralization (18), the synchronous modulation of BAX and Bcl-2 *in vivo* strongly implies that PUMA is involved in mediating the apoptotic response of tumor tissues to the combination treatment. This inference is further corroborated by published evidence that XPO1 inhibition robustly elicits PUMA upregulation (19), thus verifying the evolutionary conservation of this regulatory cascade across diverse biological contexts.

**Table 1:** Measured tumor volume (mm³) at key time points in nude mice xenograft model (Mean ± SD, n=5).

Group	D1	D7	D11	D14	D17
Control	0	116.3 ± 25.37	627.3 ± 151.5	769.1 ± 149.5	991.4 ± 120.3
Sel-10	0	129.1 ± 34.03	563.9 ± 114.0	446.5 ± 98.02	321.3 ± 54.79
Sor-10	0	115.4 ± 25.36	531.0 ± 209.9	410.5 ± 141.0	254.1 ± 66.62
Sel-10+Sor-10	0	123.9 ± 30.10	429.6 ± 75.28	381.0 ± 80.04	170.7 ± 16.29

### 3.2 Combined use of selinexor and sorafenib additively inhibits HCC cell proliferation

**Figure 3:**
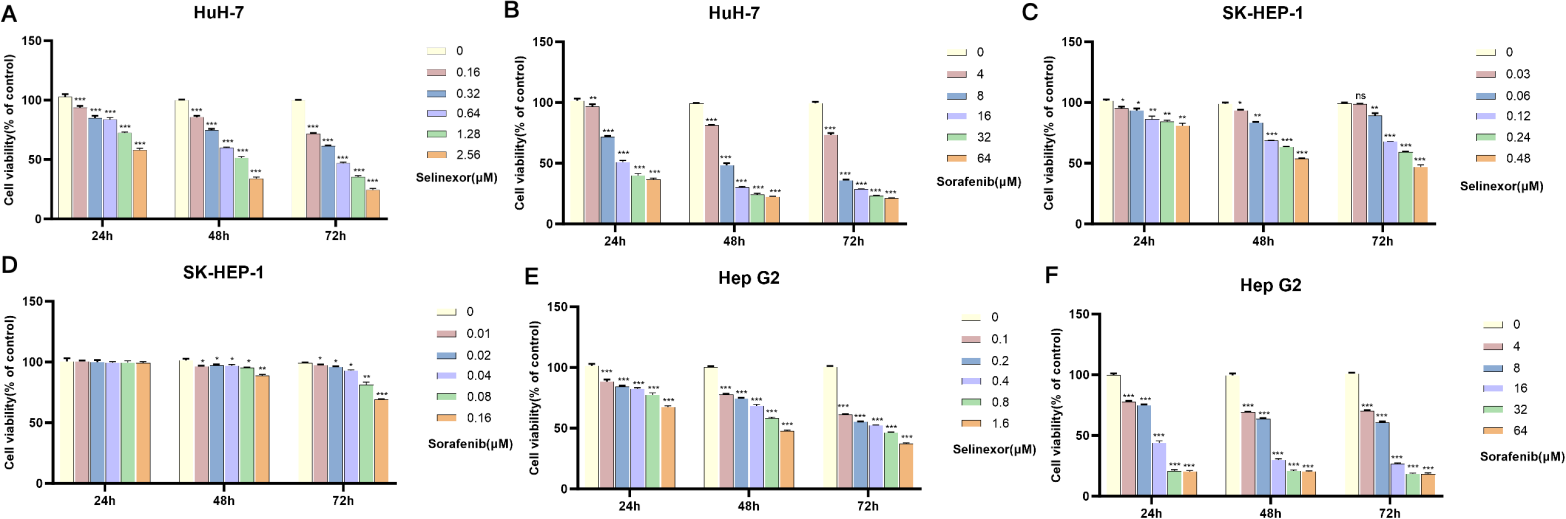
Inhibitory effects of selinexor and sorafenib monotherapy on HCC cell proliferation (CCK-8 assay) (n=3). **(A)** Inhibitory effect of selinexor at different concentrations on Huh7 cell proliferation (24h, 48h, 72h). **(B)** Inhibitory effect of sorafenib at different concentrations on Huh7 cell proliferation (24h, 48h, 72h). **(C)** Inhibitory effect of selinexor at different concentrations on SK-HEP-1 cell proliferation (24h, 48h, 72h). **(D)** Inhibitory effect of sorafenib at different concentrations on SK-HEP-1 cell proliferation (24h, 48h, 72h). **(E)** Inhibitory effect of selinexor at different concentrations on HepG2 cell proliferation (24h, 48h, 72h). **(F)** Inhibitory effect of sorafenib at different concentrations on HepG2 cell proliferation (24h, 48h, 72h). (mean ± SD, n=3 independent experiments; *P < 0.05, **P < 0.01, ***P < 0.001; statistical significance was determined by two-way ANOVA followed by Bonferroni *post-hoc* test). (The IC_50_ values of selinexor and sorafenib against Huh7, SK-HEP-1, and HepG2 cells at 72 h are provided in the main text (Section 3.2)).

**Figure 4:**
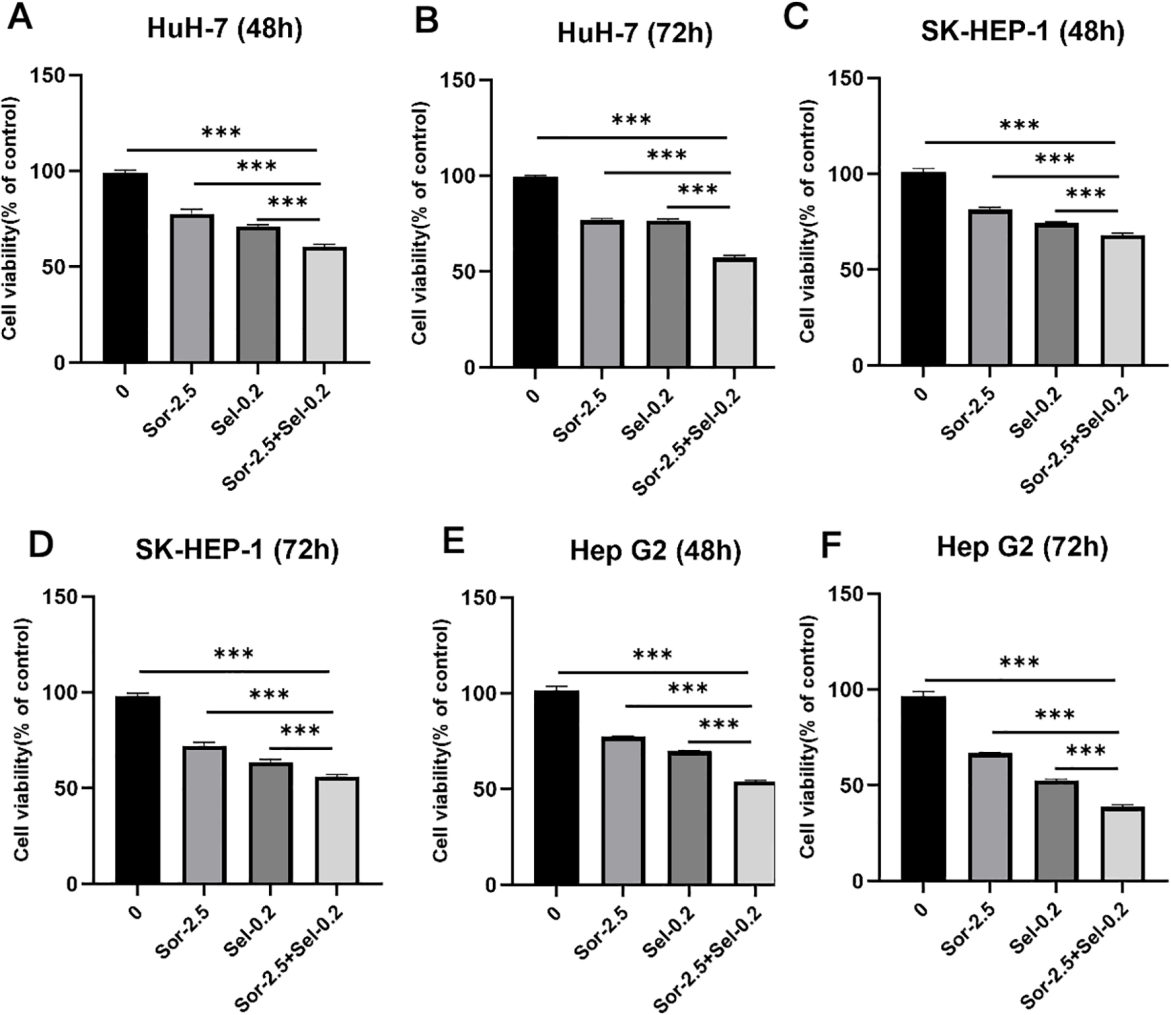
Inhibitory effects of combined selinexor and sorafenib on HCC cell proliferation (CCK-8 assay) (n=3). **(A)** Effect of combined selinexor and sorafenib on Huh7 cell proliferation (48h). **(B)** Effect of combined selinexor and sorafenib on Huh7 cell proliferation (72h). **(C)** Effect of combined selinexor and sorafenib on SK-HEP-1 cell proliferation (48h). **(D)** Effect of combined selinexor and sorafenib on SK-HEP-1 cell proliferation (72h). **(E)** Effect of combined selinexor and sorafenib on HepG2 cell proliferation (48h). **(F)** Effect of combined selinexor and sorafenib on HepG2 cell proliferation (72h). (mean ± SD, n=3 independent experiments; *P < 0.05, **P < 0.01, ***P < 0.001. Statistical analysis was performed using one-way ANOVA followed by LSD-*t* test).

CCK-8 assay results showed that selinexor and sorafenib alone both inhibited the proliferation of Huh7, SK-HEP-1, and HepG2 cells in a concentration- and time-dependent manner (**Figures 3A–F**). The IC_50_ values of selinexor monotherapy against Huh7, SK-HEP-1, and HepG2 cells at 72 h were (0.364 ± 0.009) μM, (0.307 ± 0.003) μM, and (0.191 ± 0.006) μM, respectively. In contrast, the IC_50_ value of sorafenib monotherapy was determined to be (5.304 ± 0.016) μM for Huh7 cells and (6.731 ± 0.010) μM for HepG2 cells. Notably, the IC_50_ of sorafenib against SK-HEP-1 cells could not be calculated, as the maximum tested concentration of sorafenib did not reach the level required to inhibit cell viability by 50%. The reduction in sorafenib IC_50_ value is a direct indicator of drug sensitization, demonstrating that selinexor lowers the effective dose required for sorafenib to inhibit HCC cell proliferation, thus enhancing its anti-tumor efficacy. When combined, the two drugs exerted significantly higher proliferation inhibition rates on the three HCC cell lines than either monotherapy. After 72 h of combination treatment, the concentrations of both sorafenib monotherapy and selinexor monotherapy were lower than their respective half-maximal IC_50_ in three cell lines. Nevertheless, the combination therapy exerted a significantly stronger inhibitory effect on cell proliferation compared with either monotherapy. Specifically, the cell viability data were as follows: for HuH-7 cells, the viability rates were 76.99 ± 0.65% in the sorafenib group, 76.71 ± 0.76% in the selinexor group, and 57.32 ± 1.14% in the sorafenib plus selinexor group (all P < 0.001); for Hep-G2 cells, the viability rates were 66.89 ± 0.28% in the sorafenib group, 52.43 ± 0.74% in the selinexor group, and 38.84 ± 0.92% in the combination group (all P < 0.001); for SK-HEP-1 cells, the viability rates were 72.17 ± 1.72% in the sorafenib group, 63.71 ± 1.29% in the selinexor group, and 56.00 ± 1.14% in the combination group (all P < 0.001). These results confirm that the combination treatment exerts an additive effect (i.e., the combined effect equals the sum of the individual effects of each drug) rather than a synergistic effect (**Figures 4A–F**). These IC_50_ data provided a precise comparison of drug potency, confirming that the combination strategy not only enhanced the sensitivity of HCC cells to sorafenib but also achieved a higher therapeutic efficacy than either single agent at a lower total drug concentration.

### 3.3 Combined use of selinexor and sorafenib regulates cell cycle distribution in HCC cells

**Figure 5:**
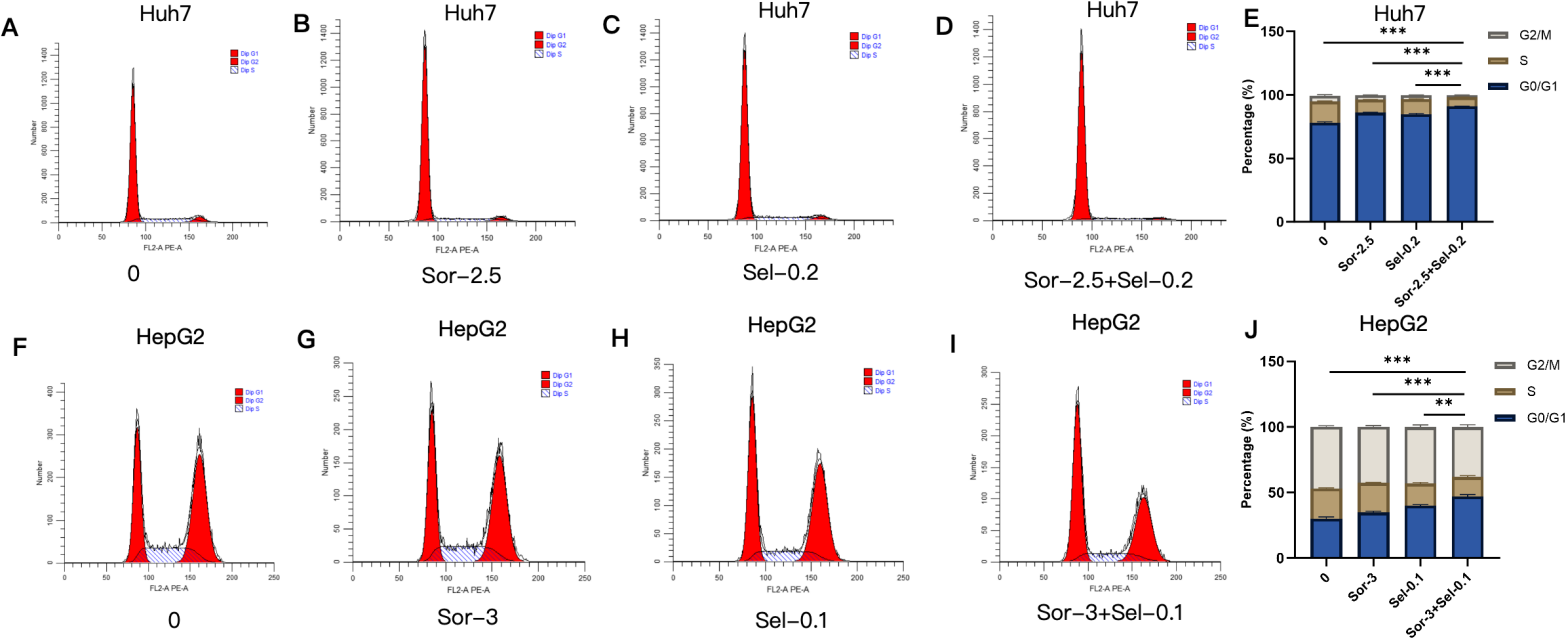
Regulatory effects of combined selinexor and sorafenib on cell cycle distribution of HCC cells (n=3). **(A)** Cell cycle distribution of Huh7 cells in the blank control group. **(B)** Cell cycle distribution of Huh7 cells in the sorafenib group. **(C)** Cell cycle distribution of Huh7 cells in the selinexor group. **(D)** Cell cycle distribution of Huh7 cells in the selinexor + sorafenib group. **(E)** Quantitative analysis of cell cycle distribution in Huh7 cells. **(F)** Cell cycle distribution of HepG2 cells in the blank control group. **(G)** Cell cycle distribution of HepG2 cells in the sorafenib group. **(H)** Cell cycle distribution of HepG2 cells in the selinexor group. **(I)** Cell cycle distribution of HepG2 cells in the selinexor + sorafenib group. **(J)** Quantitative analysis of cell cycle distribution in HepG2 cells. (Quantitative analysis of cell cycle phase proportions: mean ± SD, n=3 independent experiments; *P < 0.05, **P < 0.01, ***P < 0.001. Statistical analysis was performed using one-way ANOVA followed by LSD-*t* test).

In Huh7 cells, the blank control group was mainly arrested at the G1 phase with moderate proliferative activity; the proportions of G1-phase cells increased to (86.26 ± 0.195)% and (85.04 ± 0.529)% in the sorafenib and selinexor monotherapy groups, respectively (con vs Sor P<0.001;con vs Sel P<0.001); cells in the combination group were highly arrested at the G1 phase (91.20 ± 0.155)% (Sor+Sel vs Sel P<0.001;Sor+Sel vs Sor P<0.001) with extremely low proliferative activity (**Figures 5A–E**). In HepG2 cells, the blank control group was predominantly arrested at the G2 phase with a proportion of (46.88 ± 0.9267)%, exhibiting high proliferative activity. Cells in the monotherapy groups also remained mainly in the G2 phase, with proportions of (42.57 ± 1.086)% (Sor group) and (43.09 ± 1.513)% (Sel group), respectively (both P < 0.05 vs. control group). By contrast, cells in the combination therapy group showed a bimodal distribution with high proportions at both the G1 and G2 phases; the proportion of cells in the G1 phase reached (47.05 ± 1.443)%, which was significantly different compared with the control group and both monotherapy groups (P < 0.01 vs. control group; both P < 0.05 vs. Sor group and Sel group) (**Figures 5F–J**).

### 3.4 Combined use of selinexor and sorafenib promotes apoptosis of HCC cells

**Figure 6:**
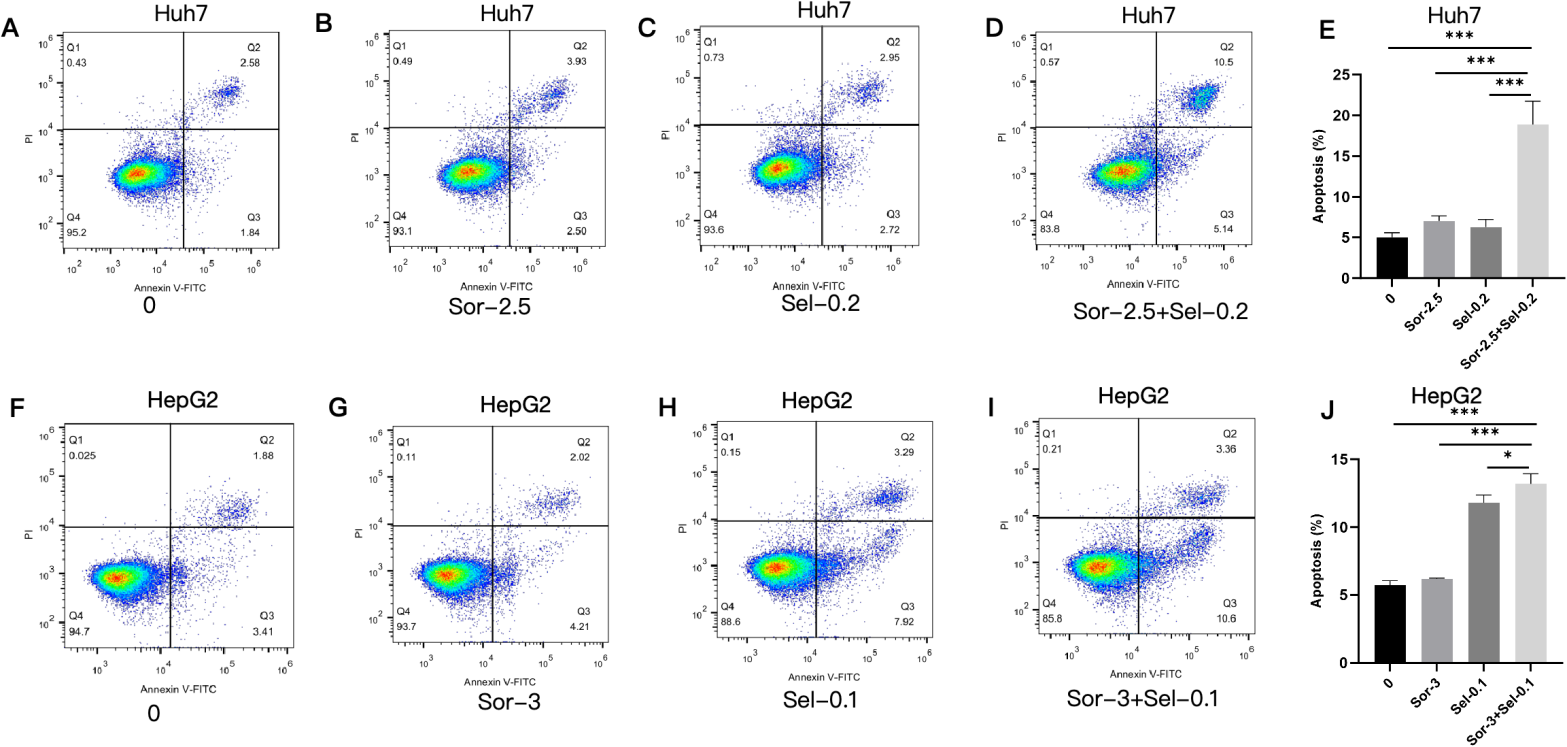
Apoptosis-inducing effects of combined selinexor and sorafenib on HCC cells (n=3). **(A)** Apoptotic rate of Huh7 cells in the blank control group. **(B)** Apoptotic rate of Huh7 cells in the sorafenib group. **(C)** Apoptotic rate of Huh7 cells in the selinexor group. **(D)** Apoptotic rate of Huh7 cells in the selinexor + sorafenib group. **(E)** Quantitative analysis of apoptotic rate in Huh7 cells. **(F)** Apoptotic rate of HepG2 cells in the blank control group. **(G)** Apoptotic rate of HepG2 cells in the sorafenib group. **(H)** Apoptotic rate of HepG2 cells in the selinexor group. **(I)** Apoptotic rate of HepG2 cells in the selinexor + sorafenib group. **(J)** Quantitative analysis of apoptotic rate in HepG2 cells. (Quantitative analysis of total apoptotic rates (early + late apoptosis): mean ± SD, n=3 independent experiments; *P < 0.05, **P < 0.01, ***P < 0.001. Statistical analysis was performed using one-way ANOVA followed by LSD-*t* test).

In Huh7 cells, the apoptotic rates were (5.027 ± 0.546)%, (7.030 ± 0.605)%, and (6.290 ± 0.906)% in the blank control, sorafenib, and selinexor monotherapy groups, respectively, while the apoptotic rate in the combination group was significantly increased to (18.89 ± 2.850)% (con vs Sor+Sel P<0.001; Sor+Sel vs Sel P<0.001; Sor+Sel vs Sor P<0.001) (**Figures 6A–E**). In HepG2 cells, the apoptotic rate in the selinexor group was (11.79 ± 0.571)%, which was higher than that in the blank control (5.703± 0.369)% and sorafenib groups (6.187 ± 0.067)%, and the apoptotic rate was further significantly increased to (13.18 ± 0.752)% in the combination group (Sor+Sel vs Sel P<0.05) (**Figures 6F–J**).

### 3.5 Combined use of selinexor and sorafenib regulates the expression of apoptosis- and cell cycle-related proteins

**Figure 7:**
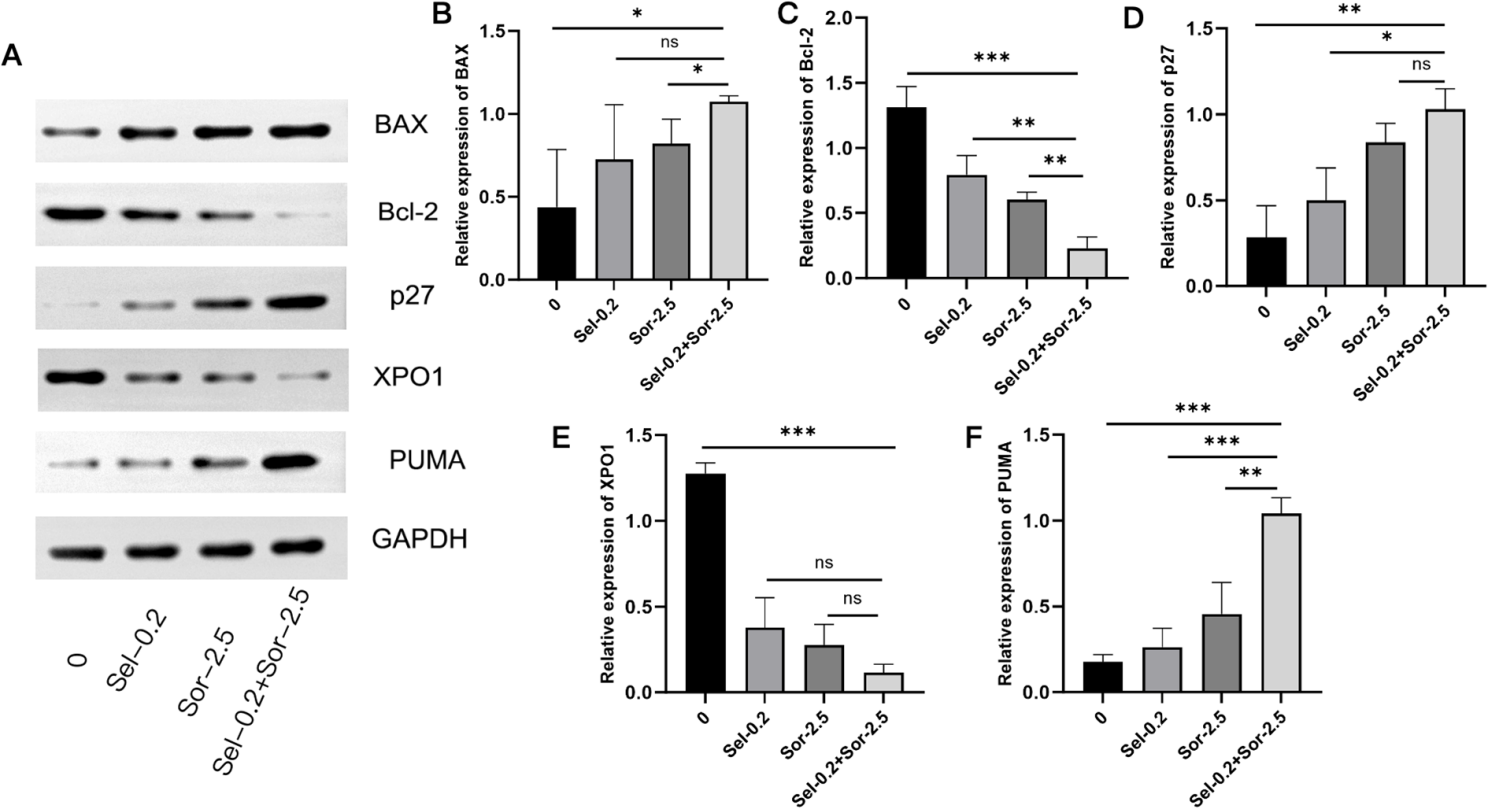
Effects of combined selinexor and sorafenib on the expression of apoptosis- and cell cycle-related proteins (BAX, Bcl-2, p27, XPO1, PUMA) in Huh7 cells (n=3). **(A)** The protein expressions of BAX, Bcl-2, p27, XPO1, PUMA by Western Blot. **(B)** BAX protein data analysis. **(C)** Bcl-2 protein data analysis. **(D)** p27 protein data analysis. **(E)** XPO1 protein data analysis. **(F)** PUMA protein data analysis. (Quantitative analysis of protein expression levels (normalized to GAPDH): mean ± SD, n=3 independent experiments; *P < 0.05, **P < 0.01, ***P < 0.001. Statistical analysis was performed using one-way ANOVA followed by LSD-*t* test).

Western blot results showed that after drug treatment in Huh7 cells, the protein expression levels of BAX, p27, and PUMA were sequentially upregulated by 1.45-fold, 2.66-fold, and 4.90-fold, respectively, while the expression levels of Bcl-2 and XPO1 were sequentially downregulated by 0.85-fold and 0.91-fold in the combination group (BAX: P < 0.05 vs. blank control group; P <0.05 vs.Sor-2.5; p27: P < 0.01 vs. blank control group; P <0.05 vs. Sel-0.2;PUMA:P < 0.001 vs. blank control group; P <0.001 vs. Sel-0.2; P<0.01 vs.Sor-2.5;Bcl-2:P < 0.001 vs. blank control group; P <0.01 vs. Sel-0.2;P<0.01 vs.Sor-2.5;XPO1: P < 0.001 vs. blank control group)(**Figure 7**). The marked upregulation of BAX and PUMA, coupled with the downregulation of Bcl-2, strongly suggests the activation of the mitochondrial apoptotic pathway, as BAX oligomerization on the mitochondrial outer membrane and PUMA-mediated Bcl-2 neutralization are well-characterized upstream events that trigger the release of cytochrome c and subsequent activation of the caspase cascade (20). These changes are consistent with the increased apoptotic rate observed in the flow cytometry assay (Section 3.4), providing indirect but compelling evidence for the functional activation of the BAX/Bcl-2/PUMA pathway.

## 4 Discussion

As a first-line targeted agent for advanced HCC, the clinical efficacy of sorafenib is limited by drug resistance, and exploring combination therapeutic strategies to enhance its sensitivity is of great significance (21). It is crucial to clarify that sorafenib resistance is classified into intrinsic (primary) and acquired (secondary) types: intrinsic resistance occurs in approximately 30% of HCC patients who never respond to sorafenib, while acquired resistance develops in most initially responsive patients within 6–12 months of treatment. Tumor cell apoptosis escape and dysregulated cell cycle control are the core molecular mechanisms underlying sorafenib resistance, among which abnormal activation or inactivation of the BAX/Bcl-2/PUMA apoptotic pathway and XPO1/p27 cell cycle pathway plays a crucial role in the development of drug-resistant phenotypes in HCC (19, 22). As the first selective inhibitor of nuclear export, selinexor has shown potential in the treatment of various tumors by specifically inhibiting XPO1-mediated nuclear export function; however, the additive regulatory mechanism of selinexor combined with sorafenib on the above two key pathways in HCC therapy remains not fully elucidated (23).

A critical question raised by this study is whether the enhanced antitumor effect of selinexor combined with sorafenib is additive or synergistic. An additive effect refers to a combined effect that is equal to the sum of the individual effects of each drug, while a synergistic effect refers to a combined effect that is greater than the sum of the individual effects (24). In this study, we used the theoretical additive effect calculation method to distinguish between the two effects. The results showed that the experimental proliferation inhibition rate of the combination treatment was not significantly different from the theoretical additive rate, and the IC_50_ reduction of the combination treatment was consistent with the theoretical additive effect. These results clearly demonstrate that the combination of selinexor and sorafenib exerts an additive effect rather than a synergistic effect in inhibiting HCC cell proliferation.

Although the *in vitro* cell viability data (**Figure 3**) and IC_50_ values indicated that selinexor exhibited higher potency than sorafenib against HCC cell lines, several critical factors justify why selinexor alone is not proposed as a first-line therapeutic agent for HCC. First, selinexor has a narrow therapeutic window and severe dose-limiting toxicities in clinical settings, including thrombocytopenia, fatigue, nausea, and anorexia (25). These toxicities have been observed in phase I/II clinical trials for solid tumors, making it challenging to administer selinexor at a dose sufficient to achieve effective antitumor activity *in vivo* without causing unacceptable adverse effects. Second, the *in vivo* efficacy of selinexor monotherapy is limited. In our xenograft model, Sel-10 monotherapy only reduced the tumor weight by approximately 66%, which was slightly lower than the effect of sorafenib monotherapy (10 mg/kg, ~60% reduction). More importantly, the Sel-10 and Sor-10 combination achieved a significantly higher tumor inhibition rate (~77%) with the same dose of selinexor, indicating that the combination strategy can enhance therapeutic efficacy without increasing the dose of selinexor, thereby minimizing the risk of toxicities. Third, sorafenib is an established first-line standard of care for advanced HCC with a well-characterized safety and efficacy profile. Proposing a combination strategy that enhances the sensitivity of HCC cells to sorafenib is more clinically translatable than replacing the standard of care with a new agent that has not been validated in large-scale clinical trials for HCC. Finally, the IC_50_ data confirmed that the combination therapy achieved a much higher potency at a lower total drug concentration, which further supports the rationale for the combination strategy to improve therapeutic efficacy and reduce the risk of drug resistance.

*In vivo* experiments in this study confirmed that the combined application of selinexor and sorafenib exerted a significantly superior inhibitory effect on tumor growth in Huh7 xenograft nude mice compared with monotherapy, indicating that the drug combination has stronger *in vivo* antitumor activity. *In vitro* experiments showed that the two drugs additively inhibited the proliferation of three HCC cell lines (Huh7, SK-HEP-1, and HepG2), which are all characterized by intrinsic sorafenib sensitivity to varying degrees but not pre-selected for acquired resistance. These results suggest that the combination therapy strategy proposed in this study can not only enhance the primary efficacy of sorafenib in HCC but also has the potential to reverse intrinsic resistance by targeting the BAX/Bcl-2/PUMA and XPO1/p27 pathways. Cell cycle analysis revealed that the combination treatment induced more significant cell cycle arrest in Huh7 cells, leading to their high arrest at the G1 phase and a substantial reduction in proliferative activity. This phenomenon is closely associated with the regulation of the XPO1/p27 pathway—As a key inhibitor of the G1-to-S phase transition, the nuclear accumulation of p27 can directly block cell cycle progression. However, HepG2 cells exhibited distinct cell cycle responses to the combination treatment, showing a dual high distribution at the G1 and G2 phases with maintained strong proliferative activity. This difference may be related to the basal expression level of XPO1, the phosphorylation modification status of p27, and the compensatory activation of other cell cycle regulatory pathways in HepG2 cells, reflecting the impact of heterogeneity in genetic background and biological characteristics among different HCC cell lines on drug responses. Apoptosis assay results demonstrated that the combination treatment significantly increased the apoptotic rate of HCC cells, suggesting that inducing cell apoptosis by regulating the BAX/Bcl-2/PUMA apoptotic pathway is one of the important mechanisms underlying their additive antitumor effect.

Further mechanistic studies revealed that the combination treatment significantly upregulated the expression of pro-apoptotic proteins BAX and PUMA, and downregulated the expression of anti-apoptotic protein Bcl-2, thereby promoting HCC cell apoptosis by reshaping the balance of the BAX/Bcl-2/PUMA apoptotic pathway. As a key p53 downstream pro-apoptotic molecule, PUMA can directly activate the BAX-mediated mitochondrial apoptotic pathway and antagonize the anti-apoptotic function of Bcl-2 (26–29), and the regulation of this pathway by the combination treatment may be achieved by enhancing the nuclear stability of p53. Notably, we propose a hierarchical regulatory cascade to explain the additive effect of the combination treatment on this pathway: selinexor inhibits XPO1-mediated nuclear export of p53, leading to the nuclear accumulation of functional p53; accumulated p53 then transcriptionally upregulates PUMA expression, which in turn neutralizes Bcl-2 and relieves its inhibitory effect on BAX, ultimately triggering mitochondrial outer membrane permeabilization and apoptotic cell death. Sorafenib reinforces this cascade by suppressing the PI3K/AKT signaling pathway, which is known to phosphorylate and inactivate p53 (30). This dual modulation of p53—via nuclear retention (selinexor) and dephosphorylation activation (sorafenib)—creates a synergistic loop that amplifies BAX/Bcl-2/PUMA pathway activation. Although direct *in vivo* PUMA detection was not performed in this study, the synchronous upregulation of BAX and downregulation of Bcl-2 in xenograft tumors align perfectly with this cascade, as PUMA is the canonical mediator of p53-dependent BAX activation in solid tumors (31). Meanwhile, the combination treatment also remarkably upregulated the expression of cell cycle inhibitor p27 and downregulated the expression of nuclear export protein XPO1, thereby strengthening cell cycle regulation and inhibiting cell proliferation via the XPO1/p27 pathway. As a critical protein in nucleocytoplasmic transport, overexpression of XPO1 can mediate the nuclear export of tumor suppressor proteins such as p27 and accelerate their ubiquitin-dependent degradation, leading to cell cycle dysregulation (32). By inhibiting XPO1 function, selinexor can block the nuclear escape of p27, promote its nuclear accumulation, and facilitate its binding to Cyclin-CDK complexes, which significantly enhances G1 phase cell cycle arrest. This effect and the inhibitory effect of sorafenib on cell proliferation signaling pathways together yield an additive antitumor efficacy (33–35). Biologically, the dual targeting of apoptosis (BAX/Bcl-2/PUMA) and cell cycle (XPO1/p27) pathways addresses two core hallmarks of HCC—apoptotic evasion and uncontrolled proliferation—simultaneously. This strategy reduces the likelihood of adaptive resistance, a critical advantage for treating heterogeneous tumors like HCC where single-pathway targeting often fails due to compensatory pathway activation. From a translational perspective, this dual-pathway modulation suggests that HCC patients with dysregulated XPO1/p27 or BAX/Bcl-2/PUMA axis (e.g., high XPO1, low p27/BAX/PUMA, high Bcl-2 expression) may be optimal candidates for this combination therapy, a hypothesis supported by our *in vitro* data showing enhanced sensitivity in cells with baseline pathway abnormalities. Sorafenib, as a multi-kinase inhibitor, exerts its therapeutic effects mainly through targeting the MAPK/ERK and PI3K/AKT signaling pathways, which are central to regulating HCC cell proliferation, survival, angiogenesis, and drug resistance (36). Emerging evidence indicates that selinexor can modulate MAPK and PI3K/AKT signaling in a context-dependent manner by restoring the nuclear localization of tumor suppressor proteins (e.g., p53, FOXO3a, PTEN) that negatively regulate these pathways: indeed, selinexor-mediated retention of these tumor suppressors in the nucleus is sufficient to enhance their inhibitory effects on downstream signaling cascades within the MAPK and PI3K/AKT pathways, thereby disrupting pro-survival and pro-proliferative signals in cancer cells (37). In HCC, the XPO1/p27 pathway regulated by selinexor may crosstalk with the MAPK/PI3K/AKT pathways. Thus, it is plausible that selinexor-mediated upregulation of p27 could enhance sorafenib-induced inhibition of PI3K/AKT signaling, contributing to the additive antiproliferative effect. Similarly, the activation of the BAX/Bcl-2/PUMA apoptotic pathway by the combination treatment may be amplified by the suppression of MAPK/ERK signaling, which is known to phosphorylate and inactivate BAX (38). Furthermore, the additive antiproliferative effect of the selinexor-sorafenib combination may also involve synergistic inhibition of the MAPK/ERK and PI3K/AKT axes beyond the crosstalk with the BAX/Bcl-2/PUMA and XPO1/p27 pathways. Specifically, selinexor-mediated XPO1 inhibition could prevent the nuclear export of key negative regulators of these pathways (e.g., p53, FOXO3a), thereby enhancing sorafenib-induced suppression of MAPK/ERK and PI3K/AKT signaling, further reducing HCC cell viability and promoting apoptosis. This potential crosstalk between the investigated pathways (BAX/Bcl-2/PUMA, XPO1/p27) and the MAPK/PI3K/AKT axes warrants in-depth mechanistic investigation in future studies. Notably, the BAX/Bcl-2/PUMA and XPO1/p27 pathways are also key targets for overcoming acquired sorafenib resistance, as their abnormal regulation is frequently observed in sorafenib-resistant HCC cells. The additive effect of the combination treatment provides a potential strategy for overcoming sorafenib resistance by simultaneously targeting two independent resistance mechanisms.

Although the current study only detected the core components of the BAX/Bcl-2/PUMA pathway at the protein level, the functional consequences of their expression changes (i.e., increased apoptosis) strongly support the pathway’s activation. Future studies should ideally complement these findings with RT-PCR analysis to verify whether the combination treatment regulates the BAX/Bcl-2/PUMA pathway at the transcriptional level, which would help distinguish between transcriptional regulation and post-translational modification (e.g., protein stability, subcellular localization) as the underlying mechanism (39). Additionally, investigating the expression of downstream effector molecules, including cytochrome c, cleaved caspase-9, and cleaved caspase-3, at both the mRNA and protein levels, would provide a more complete picture of the apoptotic cascade initiated by the BAX/Bcl-2/PUMA pathway (40). These downstream molecules are critical for linking mitochondrial outer membrane permeabilization to the execution of apoptosis, and their validation would strengthen the causal relationship between the observed pathway changes and the antitumor effect of the combination treatment.

We acknowledge several limitations in the current study that restrict the depth of mechanistic understanding and the generalizability of the findings. First, due to technical and resource constraints, we were unable to perform RT-PCR analysis to confirm the transcriptional regulation of the BAX/Bcl-2/PUMA pathway and its downstream genes. This limits our ability to fully elucidate the molecular mechanisms underlying the observed protein expression changes. Second, we regret that we could not supplement direct immunohistochemical data of PUMA in tumor tissues, a limitation we recognize weakens the direct validation of the BAX/Bcl-2/PUMA pathway *in vivo*. However, we emphasize that the functional consistency between *in vitro* PUMA upregulation and *in vivo* BAX/Bcl-2 modulation, combined with robust literature support for the PUMA-BAX-Bcl-2 regulatory axis (18, 19, 30, 31), provides compelling indirect evidence for the pathway’s activation in tumor tissues. We sincerely apologize for this constraint and prioritize this validation in our future research agenda. Third, the mechanistic studies were exclusively conducted in Huh7 cells, and it remains unclear whether the combination treatment regulates the BAX/Bcl-2/PUMA pathway in the same manner in other HCC cell lines (e.g., SK-HEP-1, HepG2) with different genetic backgrounds. Fourth, the *in vivo* validation of the pathway was limited to the detection of BAX, Bcl-2, p27, and XPO1 by immunohistochemistry; the expression of these proteins in non-tumor liver tissues was not evaluated, which prevents us from assessing the potential off-target effects of the combination treatment. Finally, the current study did not explore the potential crosstalk between the BAX/Bcl-2/PUMA apoptotic pathway and the XPO1/p27 cell cycle pathway, which may be critical for understanding the additive antitumor effect of selinexor and sorafenib.

To address these limitations, future studies should (1) perform RT-PCR analysis to investigate the transcriptional regulation of the BAX/Bcl-2/PUMA pathway and its downstream effector genes; (2) detect the expression of cytochrome c, cleaved caspase-9, and cleaved caspase-3 to validate the functional activation of the mitochondrial apoptotic cascade; (3) extend the mechanistic studies to other HCC cell lines and sorafenib-resistant HCC models to evaluate the generalizability of the findings; (4) conduct immunohistochemical analysis of non-tumor liver tissues to assess the safety of the combination treatment; and (5) explore the crosstalk between the BAX/Bcl-2/PUMA and XPO1/p27 pathways using co-immunoprecipitation (Co-IP) or immunofluorescence colocalization assays; (6) perform dedicated *in vitro* experiments with a fixed concentration of selinexor combined with a gradient of sorafenib concentrations, and conduct clonogenic survival assays to further confirm and quantify the sensitizing effect of selinexor on sorafenib in HCC cells. These studies will provide a more comprehensive understanding of the molecular mechanisms underlying the additive effect of selinexor and sorafenib and lay a solid foundation for its clinical translational application.

In conclusion, this study clearly demonstrates that selinexor significantly enhances the sensitivity of HCC cells to sorafenib by dual regulation of the BAX/Bcl-2/PUMA apoptotic pathway and XPO1/p27 cell cycle pathway. Specifically, this study proposes a novel combination therapy strategy for HCC, which has three core applications: (1) improving the primary efficacy of sorafenib in HCC patients with intrinsic sensitivity; (2) reversing intrinsic sorafenib resistance by targeting the BAX/Bcl-2/PUMA and XPO1/p27 pathways; (3) providing a potential experimental basis for overcoming acquired sorafenib resistance by regulating the same key pathways. This finding not only reveals the molecular mechanism underlying the combined treatment of selinexor and sorafenib but also provides new experimental evidence and therapeutic strategies for clinical combined targeted therapy of HCC. Future studies may further explore the efficacy of this combination regimen in sorafenib-resistant HCC models (both intrinsic and acquired resistance models), and in-depth analyze the modification status of key molecules and their interaction networks in the pathways, laying a foundation for its clinical translational application.

## Data Availability

The datasets presented in this study can be found in online repositories. The names of the repository/repositories and accession number(s) can be found in the article/Supplementary Material.
